# Hormone-Dependent Expression of a Steroidogenic Acute Regulatory Protein Natural Antisense Transcript in MA-10 Mouse Tumor Leydig Cells

**DOI:** 10.1371/journal.pone.0022822

**Published:** 2011-08-01

**Authors:** Ana Fernanda Castillo, Jinjiang Fan, Vassilios Papadopoulos, Ernesto J. Podestá

**Affiliations:** 1 Department of Human Biochemistry, School of Medicine, Instituto de Investigaciones Moleculares de Enfermedades Hormonales Neurodegenerativas y Oncológicas (IIMHNO), University of Buenos Aires, Buenos Aires, Argentina; 2 Department of Medicine and The Research Institute of the McGill University Health Centre, McGill University, Montreal, Quebec, Canada; University of Cambridge, United Kingdom

## Abstract

Cholesterol transport is essential for many physiological processes, including steroidogenesis. In steroidogenic cells hormone-induced cholesterol transport is controlled by a protein complex that includes steroidogenic acute regulatory protein (StAR). *Star* is expressed as 3.5-, 2.8-, and 1.6-kb transcripts that differ only in their 3′-untranslated regions. Because these transcripts share the same promoter, mRNA stability may be involved in their differential regulation and expression. Recently, the identification of natural antisense transcripts (NATs) has added another level of regulation to eukaryotic gene expression. Here we identified a new NAT that is complementary to the spliced *Star* mRNA sequence. Using 5′ and 3′ RACE, strand-specific RT-PCR, and ribonuclease protection assays, we demonstrated that *Star* NAT is expressed in MA-10 Leydig cells and steroidogenic murine tissues. Furthermore, we established that human chorionic gonadotropin stimulates *Star* NAT expression via cAMP. Our results show that sense-antisense *Star* RNAs may be coordinately regulated since they are co-expressed in MA-10 cells. Overexpression of *Star* NAT had a differential effect on the expression of the different *Star* sense transcripts following cAMP stimulation. Meanwhile, the levels of StAR protein and progesterone production were downregulated in the presence of *Star* NAT. Our data identify antisense transcription as an additional mechanism involved in the regulation of steroid biosynthesis.

## Introduction

Steroid hormones are essential for maintaining normal homeostasis and reproductive capability. Biosynthesis of all steroid hormones starts in the mitochondrion with conversion of cholesterol into pregnenolone by the cholesterol side-chain cleavage enzyme cytochrome P450 (P450scc; CYP11A1) [1]. Transport of cholesterol from the outer to inner mitochondrial membrane, where the conversion to pregnenolone occurs, constitutes the rate-limiting step of steroidogenesis [2], [3]. This trophic hormone-regulated step involves the formation of a macromolecular signaling complex that includes the outer mitochondrial membrane-localized translocator protein (TSPO, 18 kDa), TSPO-associated protein PAP7 (ACBD3), the regulatory α subunit of cAMP-dependent protein kinase (PRKARIα), steroidogenic acute regulatory protein (StAR; STARD1), the voltage-dependent anion channel (VDAC), and extracellular signal-regulated kinases (ERK 1/2 or MAPK3) and their upstream activator (MEK1/2) [4], [5].

In the adrenal and gonads, regulation of steroidogenesis is mediated partially by mechanisms that enhance the transcription, translation, and/or activity of StAR [6], [7]. Studies have demonstrated that regulation of StAR expression is complex, involving interaction between a diversity of hormones/factors and multiple signaling pathways [8], [9]. Synthesized as a 37-kDa precursor molecule, StAR is imported into mitochondria, where it is cleaved to generate a 30-kDa mature form [10]–[13]. To render this protein fully active in its capacity to support cholesterol transfer, StAR phosphorylation by cAMP-protein kinase A (PKA) [14] and ERK1/2 [5], [15] is required. Moreover, numerous transcription factors have been identified to bind the *Star* promoter and mediate transcription of this gene [16]. Another important regulatory mechanism of *Star* transcription involves acetylation and methylation of histones bound to the *Star* promoter [17], [18].

In addition, post-transcriptional mechanisms, such as polyadenylation, also regulate *Star* mRNA. Rodent steroidogenic cells express two main transcripts (1.6- and 3.5-kb), as well as a minor 2.8-kb form [6], [19], [20], due to differential polyadenylation in exon 7. These transcripts share the same 5′-untranslated region (5′-UTR) and open reading frame, differing only in their 3′-UTR. Thus a single protein is synthesized from all of them [20]. Mouse, rat, bovine, and human *Star* possess similar polyadenylation sites that yield equivalent alternative transcripts [21]–[23]. The synthesis and stability of the two predominant mRNAs are differentially regulated. In different rodent steroidogenic cell types, the 3.5-kb *Star* transcript is preferentially synthesized relative to the 1.6-kb mRNA after cAMP stimulation and then preferentially degraded after removal of the stimulus [24]. The fact that these transcripts share the same promoter suggests that mRNA stability is a critical regulatory mechanism of *Star*. Most genes that are controlled at the level of mRNA stability are involved in acute cellular responses to stimuli, such as early response genes, cytokines, and inflammatory mediators [25]–[28]. Within its extended 3′-UTR, the 3.5-kb *Star* form contains a region containing AU-rich elements (AUREs) and a sequence called the basal instability region (BIR) [19]. Studies have shown that the zinc finger protein TIS11b binds to these AUREs to enhance turnover of the 3.5-kb *Star* mRNA. A cAMP-stimulated AURE-independent mechanism that targets selective turnover of the 3.5-kb *Star* mRNA has also been suggested [29]. In addition, the 3′-UTR of the rodent *Star* gene also reveals putative cytoplasmic polyadenylation elements (CPEs) flanking one of the distal poly(A) signals [7]. Recruitment of CPE-binding proteins (CPEBs) to cis-elements in the 3′-UTR of mRNAs can modulate their translation in response to different stimuli [30]. Alternatively, longer 3′-UTRs frequently possess sites for microRNA (miRNA)-targeted degradation [31]. Although the impact of miRNAs on StAR expression has yet to be examined, prospective miRNA sites have already been identified within the 3′-UTR of rodent *Star* mRNA [19], [32]. While evidence for differences in StAR protein expression due to the distinct mRNAs have not been reported, additional regulatory options resulting from differential mRNA stability is most likely important in this rapid response.

Recently, the discovery of natural antisense transcripts (NATs) has added an additional level of regulation to gene expression. NATs, also named endogenous antisense transcripts, are single-stranded RNAs that are complementary to mRNA sequences (i.e., sense transcripts) [33]. These molecules can modulate the expression of sense transcripts or influence sense mRNA processing and stability [34]. Genome-wide transcriptional analyses have revealed extensive antisense transcription [35]–[37]. Although the function of most NATs remains undetermined, increasing experimental evidence indicates their involvement in gene regulation [38], [39], including genomic imprinting, alternative splicing, X inactivation, mRNA stability, translational regulation, RNA export, DNA methylation, and histone modification [40]. NATs are also involved in controlling developmental processes, adaptation to various stresses, and responses to viral infection [33], [41], [42]. These endogenous antisense transcripts may influence gene expression by interacting directly with the sense transcripts from which they are derived or affecting other targets that may be involved in mRNA transcription, maturation, transport, and/or translation [43], [44].

As mentioned above, regulation of StAR protein is a complex process. Given the functional diversity of NATs and growing evidence of their regulatory impact on protein expression, we investigated whether antisense transcription plays a role in modulating StAR protein expression and thus steroidogenesis. We identified a novel NAT that is perfectly complementary to *Star* mRNA. We also demonstrated its expression in MA-10 Leydig cells and steroidogenic murine tissues. Finally, we established that human chorionic gonadotropin (hCG) increases *Star* NAT expression via cAMP in MA-10 Leydig cells. Taken together, these data confirm the involvement of NATs in hormonal regulation.

## Results

### *In silico* prediction of NATs for the *Star* gene

To identify potential NATs specific to the *Star* mRNA, we performed a computational analysis by aligning the murine *Star* gene (Gene ID: 20845) with mouse expressed sequence tags (ESTs) using BLAST and the UCSC Genome Browser [45]. Homologous sequences were compared against the cDNA of the longest murine *Star* mRNA (NCBI Reference Sequence: NM_011485) and properties such as sequence orientation and localization of poly(A) signals and tails were analyzed. According to these criteria, several EST clones demonstrated transcription from the opposite direction (Fig. S1) and are potential NATs for *Star*.

### 5′ Rapid Amplification of cDNA Ends (5′ RACE) screening in MA-10 cells

**Figure 1:**
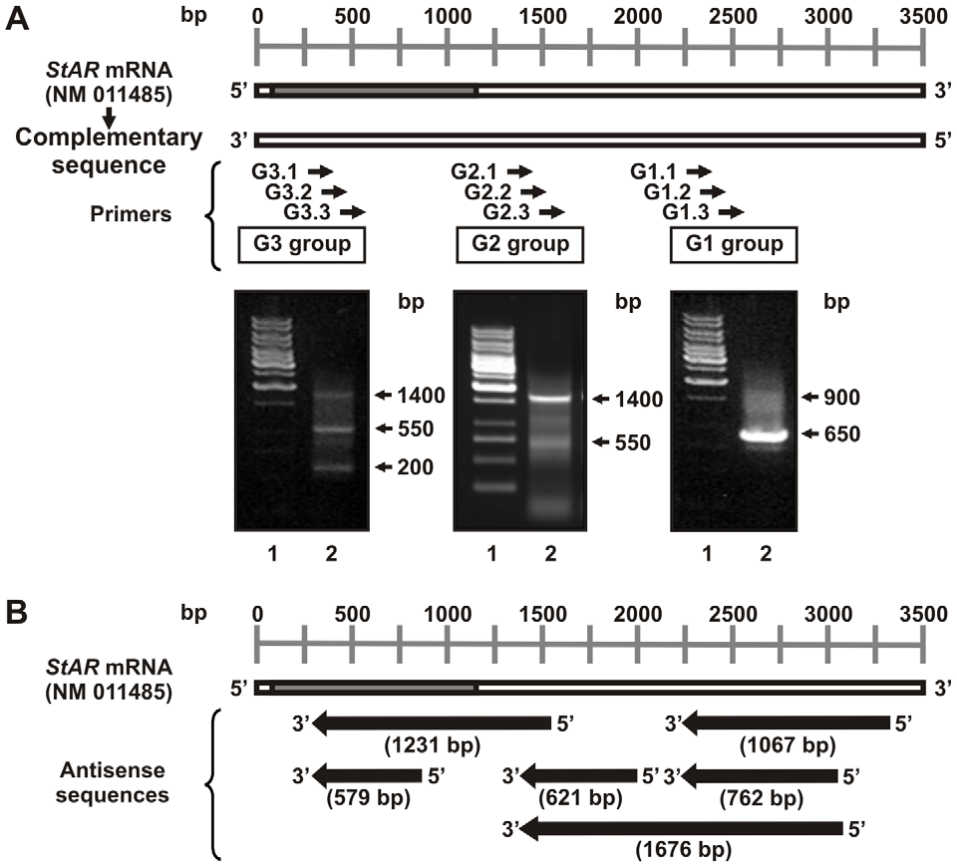
*Star* natural antisense transcript screening in MA-10 cells. Total RNA was extracted from MA-10 cells and treated with DNase I. 5′ RACE was performed using three different sets of three sequence-specific primers for RT, PCR, and nested PCR. Following agarose gel electrophoresis, bands were eluted, and cloned into the pGEM®-T Easy vector for sequencing. The results were analyzed by BLAST and Vector NTITM Suite 8 software. **A**. *Upper panel.* Schematic diagram showing the relative location of the three groups of primers (G1, G2, and G3) used. *Lower panels.* Representative images of the nested PCR products generated by amplification with each primer group (lane 2). Lane 1 shows the DNA molecular weight ladder. Arrows indicate the apparent sizes (in base pairs, or bp) of the eluted bands. **B**. Schematic diagram showing the representative sequences of the resultant products. Sizes are indicated in bp. Complementarity and relative location of these sequences with the *Star* transcript sequence is indicated.

Given that 10 to 20% of EST sequences in UniGene were annotated in the wrong direction [46], reliable *in silico* screenings must be based on stringent parameters which could underestimate or exclude some genuine antisense transcripts. Nevertheless, we proceeded to experimentally validate our bioinformatics results by performing 5′ RACE experiments on total RNA isolated from the MA-10 Leydig tumor cell line. Three different groups consisting of three sequence-specific primers were used for the RT, PCR, and nested PCR analyses respectively (Fig. 1A and Table 1). Before any reaction, RNA was treated with RNase-free deoxyribonuclease I (DNase I) to eliminate any contaminating genomic DNA. Moreover, the identity of the amplified products was confirmed by sequencing in both directions. This analysis verified the presence of antisense sequences of different sizes that were completely complementary to *Star* mRNA (Fig. 1B). Many overlapped with each other, suggesting that they could be fragments of a longer, unique antisense sequence.

**Table 1:** Oligonucleotide sequences used in this study.

Reaction	Primer			Sequence (5′ – 3′)
5′ RACE	G1 Group	RT	G1.1	TGAATCGCTCCAAGTTCCAGGCCAGCT
		PCR	G1.2	CCAGCTTGGACCCCAGTGAGACCGTCT
		nested PCR	G1.3	GCCTGGCTCAGAGCTAGCAGCTTCCTTA
	G2 Group	RT	G2.1	CCAGCAGCTACGAACAGGGGATG
		PCR	G2.2	CGGGAACTGTTGTCTTACCAGCTCCAAA
		nested PCR	G2.3	ACCATCATCGTGCCGACTTCCCT
	G3 Group	RT	G3.1	TCCAGCAGGGAGAGGTGGCTATGCAGA
		PCR	G3.2	GGCTGGAAGAAGGAAAGCCAGCAGGAGA
		nested PCR	G3.3	AAGATGGTGCCAGATGTGGGCAAGGTGT
3′ RACE		PCR	G4.1	ACACCTTGCCCACATCTGGCACCATCTT
		Nested PCR	G4.2	CCTGCTGGCTTTCCTTCTTCCAGCCTT
*Star* NAT amplification	RT		G3.1	TCCAGCAGGGAGAGGTGGCTATGCAGA
	PCR		Fw.1	CCCTCGCTCACCTTAAAGCACCG
			G3.2	GGCTGGAAGAAGGAAAGCCAGCAGGAGA
	nested PCR		Fw.2	GCAAATGATGGGGGTTACCCACA
			G3.3	AAGATGGTGCCAGATGTGGGCAAGGTGT
*Star* NAT full amplification	RT		Rv.RT	TTATCTCAAGTGATGATGCACAGCCTT
	PCR		Fw.1	CCCTCGCTCACCTTAAAGCACCG
			Rv.1	CTTCCACGGGAAGCATTTAAGGCA
	nested PCR		Fw.2	GCAAATGATGGGGGTTACCCACA
			Rv.2	CATTTAAGGCAGCGCACTTGATCT
Semiquantitative RT-PCR	*Star* NAT	RT	G3.1	TCCAGCAGGGAGAGGTGGCTATGCAGA
		PCR	Fw.Nat	CCTGGCTCCCAATTTATACC
			Rv.Nat	TAGGCTGAACACCCATTAACATT
	L19	RT		Random primers (Promega)
		forward	L19.Fw	GAAATCGCCAATGCCAACTC
		reverse	L19.Rv	TCTTAGACCTGCGAGCCTCA
	*Star* Sense 1	forward	Fw.S1	AAACAGAGGTCCTTATGGCTGC
		RT/reverse	Rv.S1	GGTGAGCAATAAATATCAGTGAAGG
	*Star* Sense 2	forward	Fw.S2	TTTAATCCCAACACTCAAGTGG
		RT/reverse	Rv.S2	CCTGGAACAGGAATGTTATAGC
	*Star* Sense 3	forward	Fw.S3	GGGACGAAGTGCTAAGTAAGATGG
		RT/reverse	Rv.S3	GGTCAATGTGGTGGACAGTCC

### *Star* NAT expression in MA-10 cells

**Figure 2:**
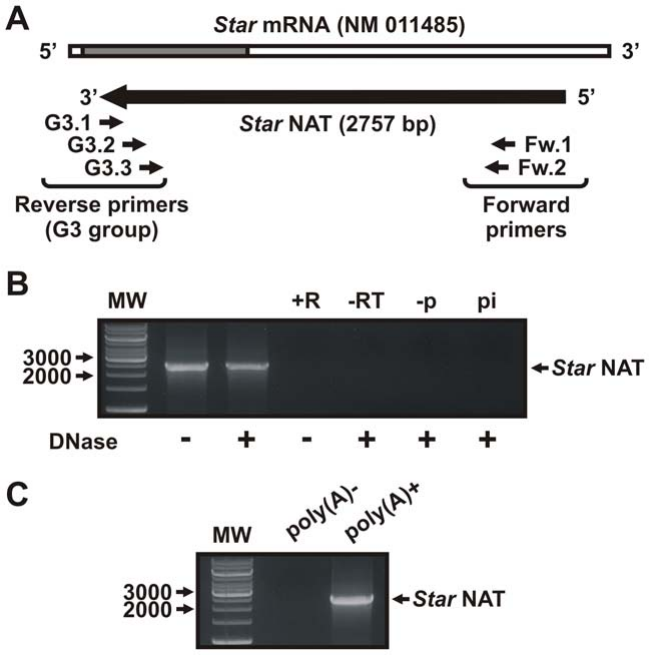
*Star* NAT expression in MA-10 cells. Total RNA was extracted from MA-10 cells and treated with DNase or RNase prior to sequence-specific reverse transcription, followed by PCR/nested PCR and sequencing. **A**. Schematic diagram showing the RT primer (G3.1), PCR primers (Fw.1 and G3.2), and nested PCR primers (Fw.2 and G3.3) used in these experiments. A schematic representation of the full *Star* NAT sequence obtained after sequencing the PCR products is shown. **B**. Representative image of the RT-nested PCR product, the identity of which was confirmed by sequencing. +R, RNase-treated RNA control; -RT, no reverse transcriptase RT-PCR control; -p, RT reaction in the absence of primer; pi, RT reaction in the presence of a non-specific primer; MW, DNA molecular weight ladder. Arrows indicate apparent sizes in bp. **C**. Poly (A)^+^ RNA was purified from total MA-10 cells RNA using oligo(dT) cellulose chromatography. After DNase I treatment, specific RT, PCR and nested PCR were conducted as indicated above. A representative image of the PCR product is shown.

To evaluate the presence of a long *Star* antisense transcript, sequence-specific RT-PCR was performed using DNase I-treated total RNA from MA-10 cells. The relative orientation of transcription was assessed by restricting which primer was present during single-strand cDNA synthesis. For this purpose, the first primer of the G3 group designed for the 5′ RACE assay was used. For the PCR and nested PCR amplifications, the remaining two primers of the G3 group were used as reverse primers. The two forward primers were designed based on the RACE results (Fig. 2A).

Cloning and sequencing the PCR product confirmed the presence of a 2757-bp transcript that was perfectly complementary to the *Star* mRNA sequence, including parts of the coding region (i.e., exons 4, 5, 6, and part of exon 7) and the 3′-UTR of the longest *Star* sense mRNA (Fig. 2B). Thus, hereafter we will refer to this transcript as the *Star* NAT.

To verify this result, several negative controls were included. A ribonuclease (RNase)-treated RNA control was analyzed to identify any RNA contamination in the reaction. In addition, RT-PCR of a DNase I-treated RNA sample without reverse transcriptase was performed. Two controls were conducted in order to evaluate the strand specificity of the reverse transcription: first, an RT reaction conducted in the absence of primer and a second RT reaction done in the presence of a non-specific primer. PCR product was not detected from these negative control reactions. Moreover, RT reactions were carried out at high temperature (i.e., 50°C) in order to avoid primer-independent cDNA synthesis [47] and to improve strand specificity in RT-PCR.

### *Star* NAT characterization

Since total RNA contains both polyadenylated [poly(A)^+^] and non-polyadenylated [poly(A)^-^] RNA molecules. To determine the class in which *Star* NAT should be categorized, MA-10 cell total RNA was divided into two fractions and analyzed in parallel by sequence-specific RT, PCR, and nested PCR following DNase I treatment. Fig. 2C shows that *Star* NAT was amplified from poly(A)^+^ but not poly(A)^−^ RNA, indicating that this transcript belongs to the class of polyadenylated RNAs. However, neither a classical polyadenylation consensus region nor a poly(A) tail was detected in the *Star* NAT sequence.

**Figure 3:**
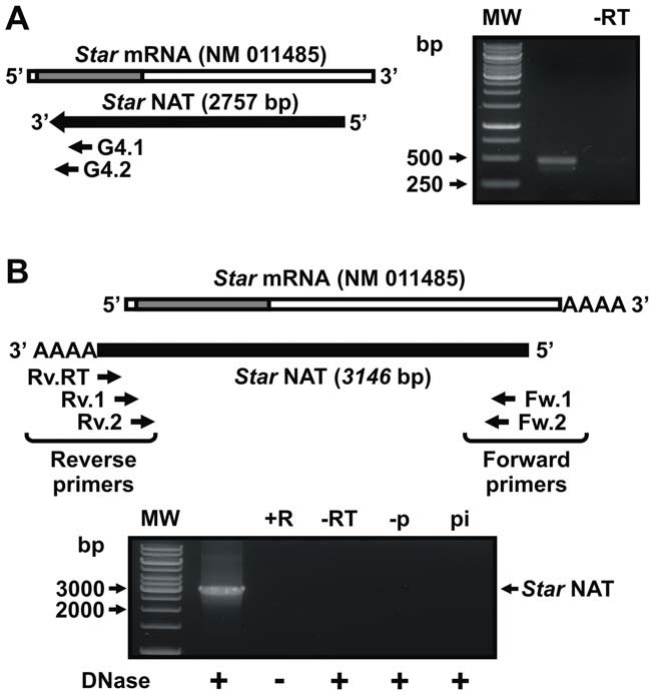
*Star* NAT 3′ end characterization. **A**. Total RNA was isolated from MA-10 cells and treated with DNase I. 3′ RACE was performed using two sequence-specific primers for PCR and nested PCR amplifications. Following agarose gel electrophoresis, bands were eluted, and cloned into the pGEM®-T Easy vector for sequencing. The results were analyzed by BLAST and Vector NTITM Suite 8 software. *Left panel.* Schematic diagram showing the relative location of the sequence-specific primers used. *Right panel.* Representative image of the nested PCR product generated. MW, DNA molecular weight ladder: -RT, no reverse transcriptase RT-PCR control. Arrows indicate the apparent sizes in bp. **B**. Total RNA extracted from MA-10 cells was treated with DNase or RNase prior to sequence-specific reverse transcription, followed by PCR/nested PCR and sequencing. *Upper panel.* Schematic diagram showing the RT primer (Rv.RT), PCR primers (Fw.1 and Rv.1), and nested PCR primers (Fw.2 and Rv.2) used in these experiments. A schematic representation of the full *Star* NAT sequence obtained after sequencing the PCR product is shown. *Lower panel.* Representative image of the RT-nested PCR product. +R, RNase-treated RNA control; -RT, no reverse transcriptase RT-PCR control; -p, RT reaction in the absence of primer; pi, RT reaction in the presence of a non-specific primer; MW, DNA molecular weight ladder. Arrows indicate apparent sizes in bp.

To evaluate *Star* NAT missing 3′end sequence, 3′ RACE experiments on DNase I treated- total RNA isolated from MA-10 cells were performed (Fig. 3A). Analysis of the amplified products showed an extension on *Star* NAT sequence of 389-bp reaching its poly(A) tail. We next performed a sequence-specific RT-PCR to confirm that this new fragment is part of a full length *Star* NAT transcript. Three primers were designed based on the results of the 3′ RACE experiments, one for the RT reaction and the remaining two as reverse primers for PCR and nested PCR amplifications (Table 1). Fig. 3B shows a PCR product that confirmed the presence of a 3146-bp transcript that was perfectly complementary to the *Star* mRNA sequence, including the 5′-UTR, the coding region and part of the 3′-UTR of the longest *Star* sense mRNA. Interestingly, the last 75-bp of *Star* NAT 3′end is complementary to the *Star* genomic sequence, beyond the 5′ end of *Star* mRNA.

To further characterize this antisense transcript, the presence of open reading frame (ORF) coding proteins was analyzed. Then, probable amino acid sequences derived from this transcript were compared against murine protein databases using BLAST. *Star* NAT does not seem to encode any known protein unlike many other antisense transcripts described in the literature [48]. We also explored the possibility of a promoter region that regulates *Star* NAT transcription; however, none was identified within the *Star* gene or its flanking genomic sequences.

### Expression of *Star* NAT in mouse tissues

**Figure 4:**
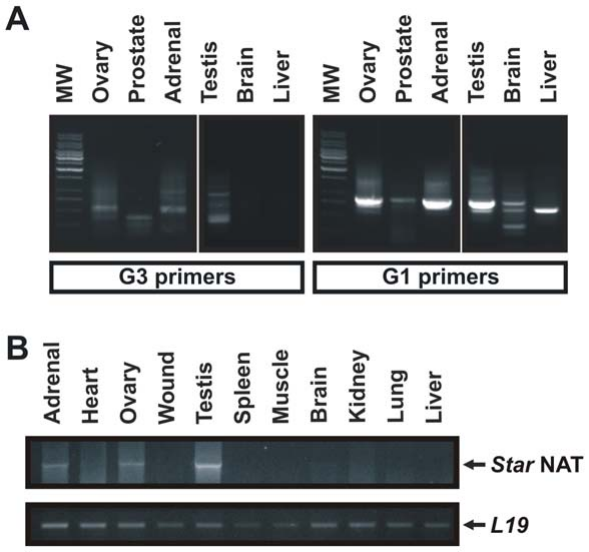
*Star* NAT expression in mouse tissues. **A**. Total RNA was extracted from different mouse tissues and treated with DNase I. 5′ RACE was performed as described in Fig. 1. A representative image of the RT-nested PCR products generated using the G1 and G3 primers is shown. MW, DNA molecular weight ladder. **B**. *Star* NAT sequence-specific reverse transcription was performed using DNase I-treated total RNA from different mouse tissues, followed by PCR/nested PCR as described in Fig. 2. *L19* mRNA, a housekeeping ribosomal protein, was used as a loading control. A representative image of the RT-nested PCR product is shown. The identity of the amplification products was confirmed by sequencing.

To further validate our observations, we studied the expression of *Star* endogenous antisense transcripts in mouse tissues. 5′ RACE experiments were performed with total DNase I-treated RNA isolated from mouse testis, ovary, adrenal gland, prostate, liver, kidney, and brain. Fig. 4A demonstrates that steroidogenic tissues, namely the ovary, adrenal gland, and testis, display a similar outcome to MA-10 cells, suggesting the presence of overlapping antisense sequences that were completely complementary to *Star* mRNA. Also, other partially complementary antisense sequences for *Star* were found in the liver, ovary, and brain.

To assess the presence of a specific long *Star* NAT in mouse tissues, sequence-specific RT, PCR, and nested PCR were conducted as described previously for MA-10 cells. A single band of the expected size was detected and the identity of this amplification product was confirmed by sequencing. Fig. 4B shows that the *Star* NAT found in the MA-10 cell line is also expressed in classical steroidogenic tissues, such as adrenal gland, ovary, testis, and brain.

### Hormonal regulation of *Star* NAT expression

**Figure 5:**
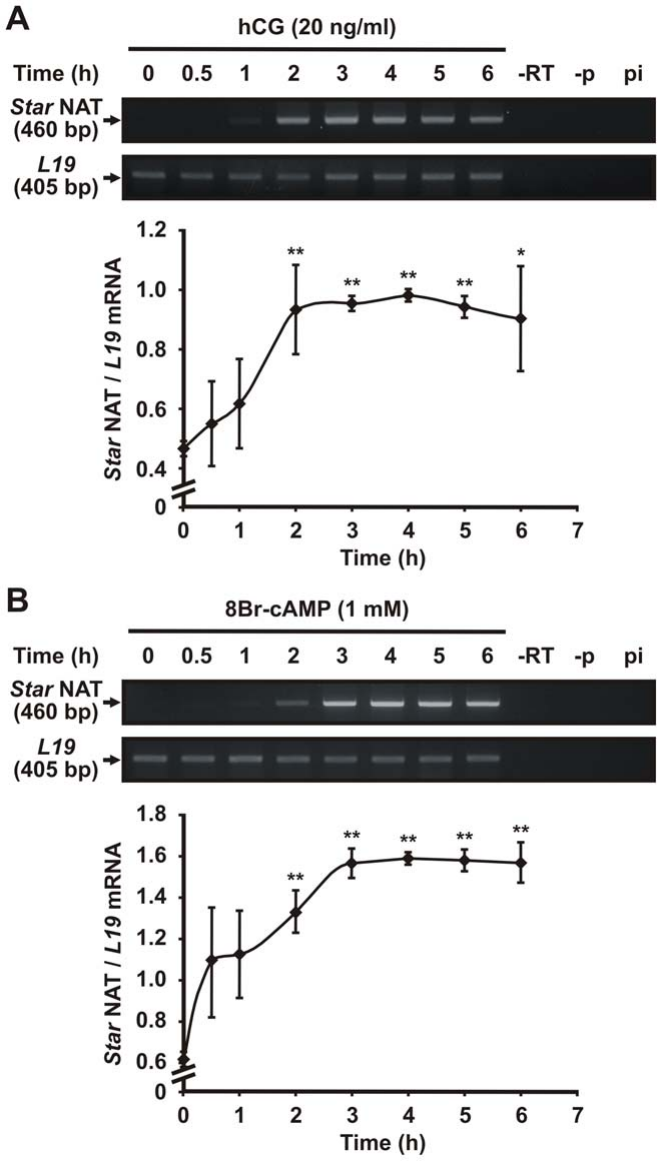
Analysis of hormone-stimulated *Star* NAT expression by RT-PCR. MA-10 cells were incubated with hCG (20 ng/ml) (**A**) or 8Br-cAMP (1 mM) (**B**) for the indicated times. Total RNA was extracted and treated with DNase I. Semi-quantitative sequence-specific RT-PCR was performed to evaluate *Star* NAT expression. *L19* mRNA expression was evaluated as a loading control. The images of representative agarose gels are shown. The sizes of the amplified products are indicated in bp. For each time point, the optical density (OD) of *Star* NAT expression was quantified and normalized to the corresponding *L19* mRNA level. Graphs show the relative expression levels of *Star* NAT. Data are presented as an average ± SD of four independent experiments. * *P*<0.05 and ** *P*<0.01 *vs.* non-stimulated cells. -RT, no reverse transcriptase RT-PCR control; -p, RT reaction in the absence of primer; pi, RT reaction in the presence of a non-specific primer.

To elucidate a possible functional role for *Star* NAT expression, hormonal regulation of this transcript was assessed. Semi-quantitative RT-PCR was performed on MA-10 cells treated with human chorionic gonadotropin (hCG) for varying times (i.e., 0 to 6 h). Fig. 5A demonstrates that hCG-induced *Star* NAT expression was time-dependent. Maximum levels were observed after 2 to 3 h of hormone stimulation. *Star* NAT expression also increased in a time-dependent manner when cells were stimulated with cAMP (Fig. 5B), with maximum levels observed 3 h after stimulation.

**Figure 6:**
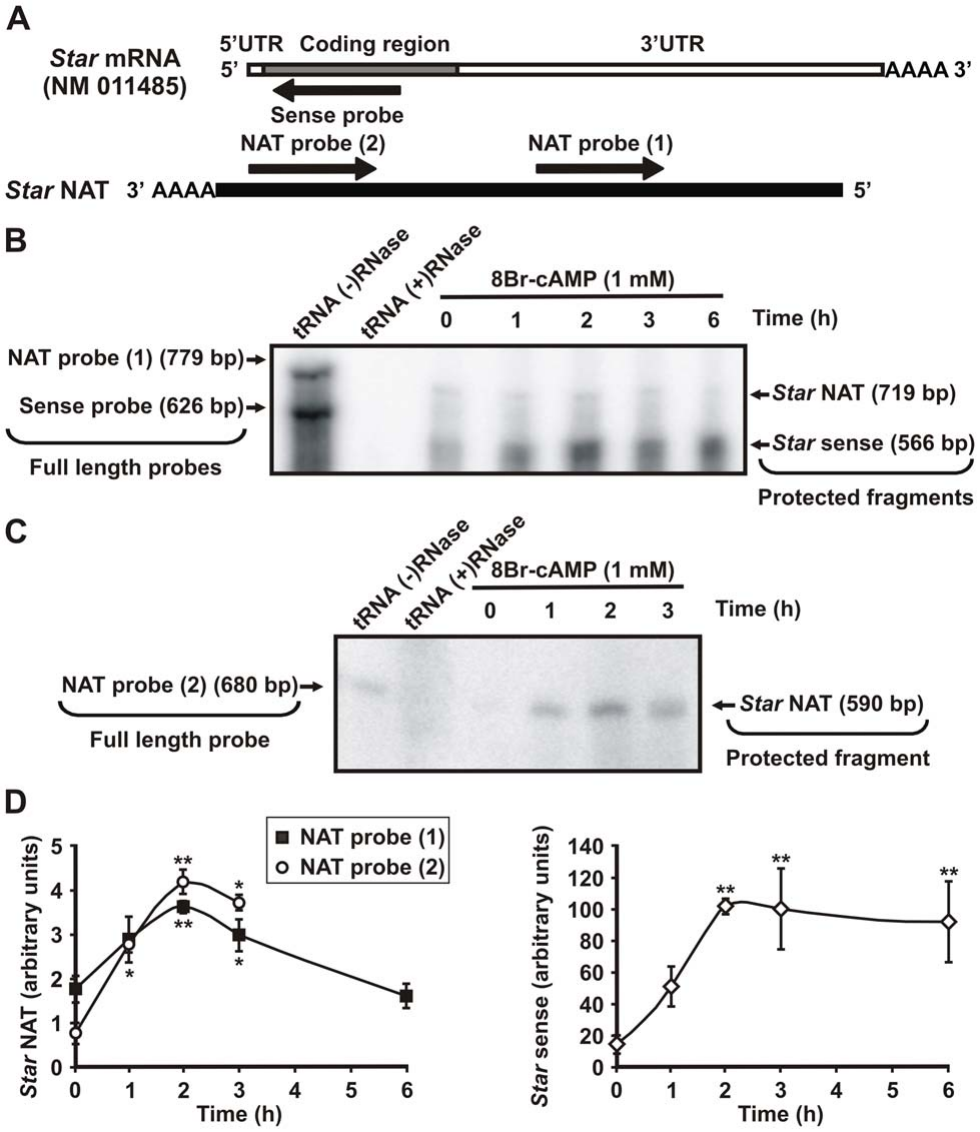
Analysis of hormone-regulated *Star* NAT expression by RNase protection assay (RPA). **A**. MA-10 cells were incubated with 8Br-cAMP (1 mM) for the indicated times. Total RNA was extracted and then treated with DNase I. Single-chain *Star* sense and *Star* NAT RNA probes were synthesized by in vitro transcription and labeled with [^32^P] UTP. A schematic diagram illustrates the riboprobes used and their complementarity to the corresponding transcript. **B**. RPA was performed using simultaneously two probes: the sense- *Star* RNA probe (Sense probe) that was complementary to 566 bp of the coding region of sense transcripts, and the NAT- *Star* RNA probe (NAT probe 1) that was complementary to 719 bp of *Star* NAT. A representative autoradiograph is shown. The negative control (tRNA (+) RNase) consisted of yeast tRNA instead of MA-10 RNA. The tRNA (−) RNase control consisted of yeast tRNA without RNase treatment and was used to visualize the full-length probes. **C**. RPA was performed using another *Star* NAT probe (NAT probe 2) that was synthesized to be complementary to 590 bp of *Star* NAT sequence in a region different from the probe used in (B). A representative autoradiograph is shown. **D**. Graphs show the quantification (OD) of *Star* sense and NAT expression levels for each time point expressed in arbitrary units. Data are presented as an average ± SD of three independent experiments. * *P*<0.05 and ** *P*<0.01 *vs.* non-stimulated cells.

To support these data, an RNase protection assay (RPA) was conducted to confirm the presence of the antisense transcript. In this experiment, MA-10 cells were incubated with 8Br-cAMP (1 mM) for varying times. Total RNA was extracted, treated with DNase, and then co-precipitated with single chain riboprobes specific to the *Star* mRNA sense strand and NAT (Fig. 6A). Hybridization and RNase A digestion were subsequently performed. Moreover, yeast tRNA with and without RNase treatment were used as controls. Fig. 6B shows that, consistent with the RT-PCR data, cAMP treatment increased *Star* NAT expression levels in a time-dependent manner. This experiment also demonstrated coordinated regulation of both sense and antisense *Star* transcripts after hormone stimulation. Since the *Star* sense probe targeted the coding region, joint expression of the three sense mRNAs (1.6-, 2.8- and 3.5-kb) was assessed in this approach. The data show that the antisense transcript exists simultaneously with its sense counterpart.

For further confirmation, this experiment was repeated using a *Star* NAT probe that was complementary to the antisense transcript in a different region, its 3′-end. Identical results were obtained with this approach (Fig. 6C), thereby validating our methodology as well as the presence and hormonal regulation of *Star* NAT via cAMP. These findings suggest that *Star* NAT may play a role in hormonal regulation of StAR protein expression and therefore steroid synthesis.

### Effect of *Star* NAT overexpression on *Star* sense transcripts, StAR protein levels, and steroid production

**Figure 7:**
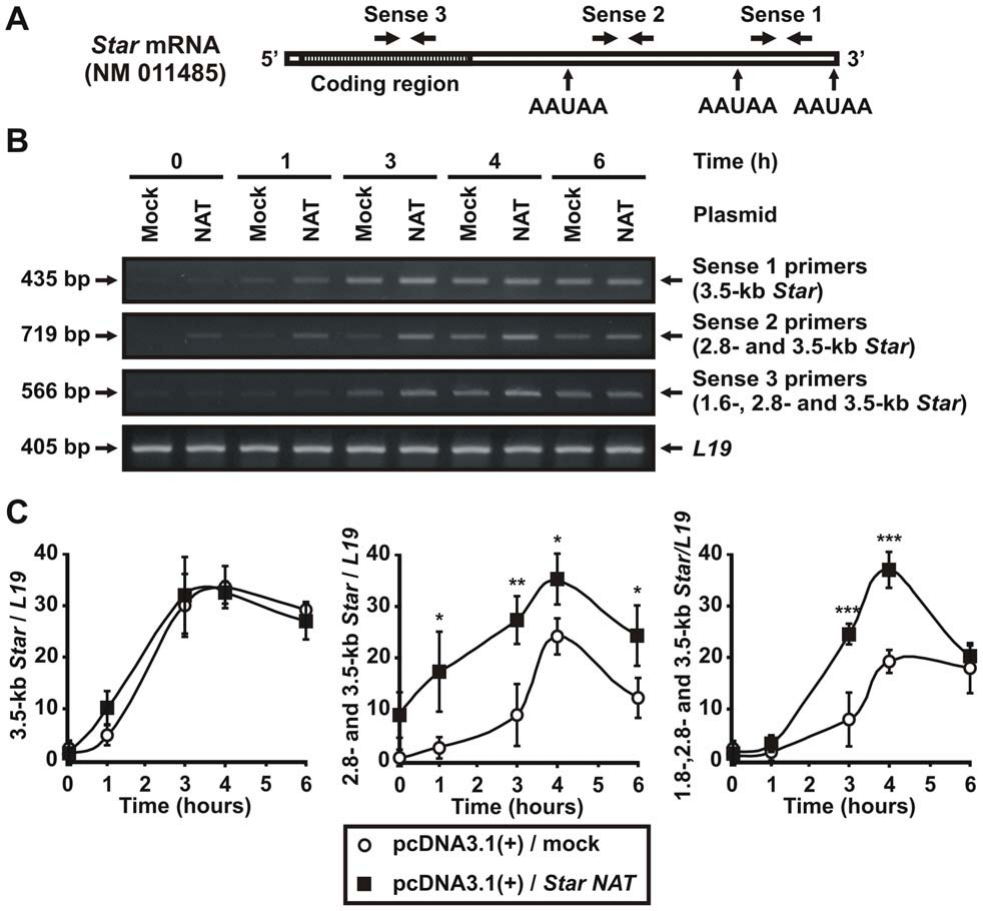
Assessment of the effect of *Star* NAT overexpression on the level of *Star* sense transcripts by RT-PCR. MA-10 cells were transiently transfected with a pcDNA3.1(+) vector expressing *Star* NAT (NAT) or an empty vector (mock) as a control. At 24 h post-transfection, the cells were stimulated with 8Br-cAMP for the indicated times. Total RNA was extracted and semi-quantitative RT-PCR of *Star* sense transcripts was performed. **A**. Schematic diagram showing the three primer pairs targeting different regions within the mouse *Star* mRNA used in these experiments. Sense 1 primers target the 3′-UTR of the longest 3.5-kb mRNA. Sense 2 primers target a region shared by the 2.8- and 3.5-kb *Star* mRNAs. Sense 3 primers target the coding region shared by all three sense transcripts. AAUAA, polyadenylation signals. **B**. Images of representative agarose gels are shown. Arrows indicate the sizes in bp of the amplified products. *L19* mRNA expression was evaluated as a loading control. **C**. For each band, the OD of the level of *Star* sense transcripts was quantified and normalized to the corresponding *L19* mRNA level. Graphs show the relative expression of *Star* sense transcripts. Data are presented as an average ± SD of three independent experiments. Data are presented as an average ± SD of three independent experiments. * *P*<0.05, ** *P*<0.01 and *** *P*<0.001 *vs.* mock-transfected cells stimulated during the corresponding time.

To investigate the functional role of *Star* NAT expression in steroid synthesis, MA-10 cells were transiently transfected with a pcDNA3.1(+) vector expressing *Star* NAT. At 24 h post-transfection, cells were stimulated with 8Br-cAMP for varying times. Total RNA, mitochondria, and culture media were harvested and analyzed. First, the expression levels of *Star* sense transcripts were determined by semi-quantitative RT-PCR. Since the three *Star* mRNAs differ only within their 3′-UTR, discriminating between them was difficult. Thus, three primer pairs targeting different regions of mouse *Star* mRNA were used (Fig. 7A and Table 1). This analysis revealed that *Star* NAT overexpression had a differential effect on *Star* sense transcripts expression (Fig. 7B and C). While no significant variation was found in the level of the 3.5-kb transcript, a dramatic increase was observed when the 2.8- and 3.5-kb forms were determined together, indicating that *Star* NAT overexpression increases 8Br-cAMP-induced expression of the 2.8-kb mRNA. When the levels of all three *Star* sense transcripts were examined jointly, this increase was also detected. Nevertheless, this experiment did not allow us to evaluate the contribution, if any, of the 1.6-kb mRNA in the observed effect.

**Figure 8:**
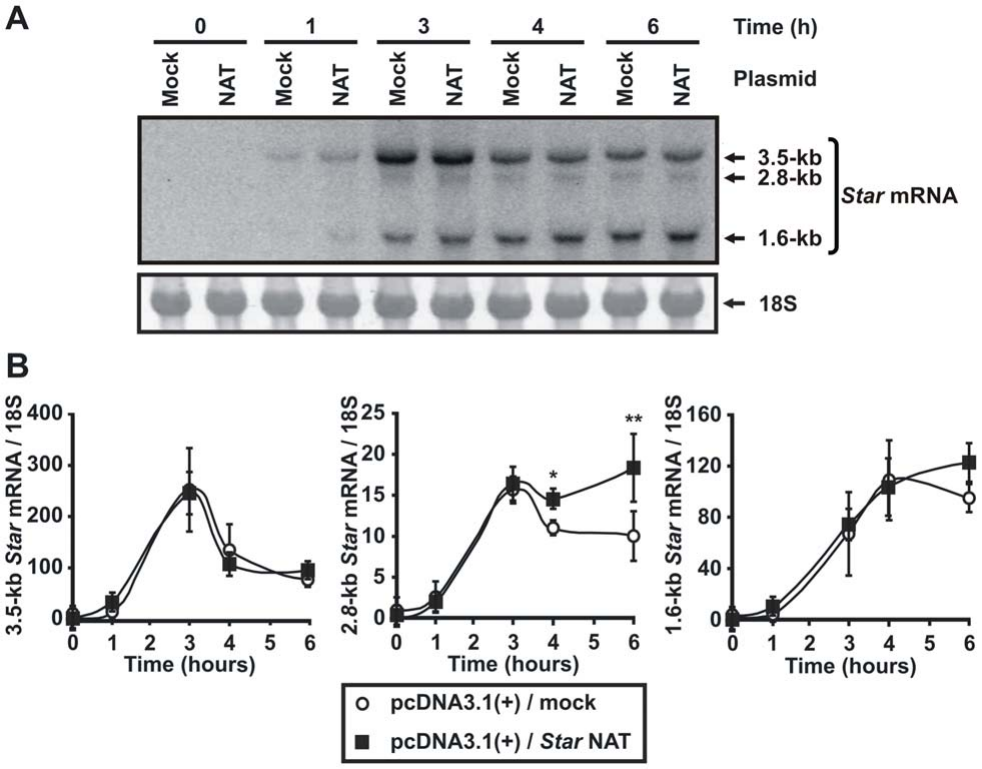
Assessment of the effect of *Star* NAT overexpression on the level of *Star* sense transcripts by Northern blot analysis. MA-10 cells were transiently transfected with a pcDNA3.1(+) vector expressing *Star* NAT (NAT) or an empty vector (mock) as a control. At 24 h post-transfection, the cells were stimulated with 8Br-cAMP for the indicated times. Total RNA was extracted and northern blotting was conducted. **A**. An image of a representative northern blot is shown. A single-chain *Star* sense riboprobe was used in this assay. 18S rRNA was used as a loading control. **B**. For each band, the OD of the expression level of each *Star* transcript was quantified and normalized to the corresponding 18S RNA level. Graphs show the relative levels of *Star* sense transcripts. Data are presented as an average ± SD of three independent experiments. * *P*<0.05 and ** *P*<0.01 *vs.* mock-transfected cells stimulated during the corresponding time.

Thus, to address this and establish the selectivity of *Star* NAT, the expression levels of *Star* sense transcripts in transfected MA-10 cells was determined by Northern blot analysis. Although relatively less sensitive, this assay enabled us to evaluate each mRNA individually. The data confirmed that *Star* NAT overexpression increased 8Br-cAMP-promoted 2.8-kb and 1.6-kb *Star* mRNAs expression after 4 and 6 h of stimulation respectively, while expression of the 3.5-kb transcript was unaffected (Fig. 8A and B).

**Figure 9:**
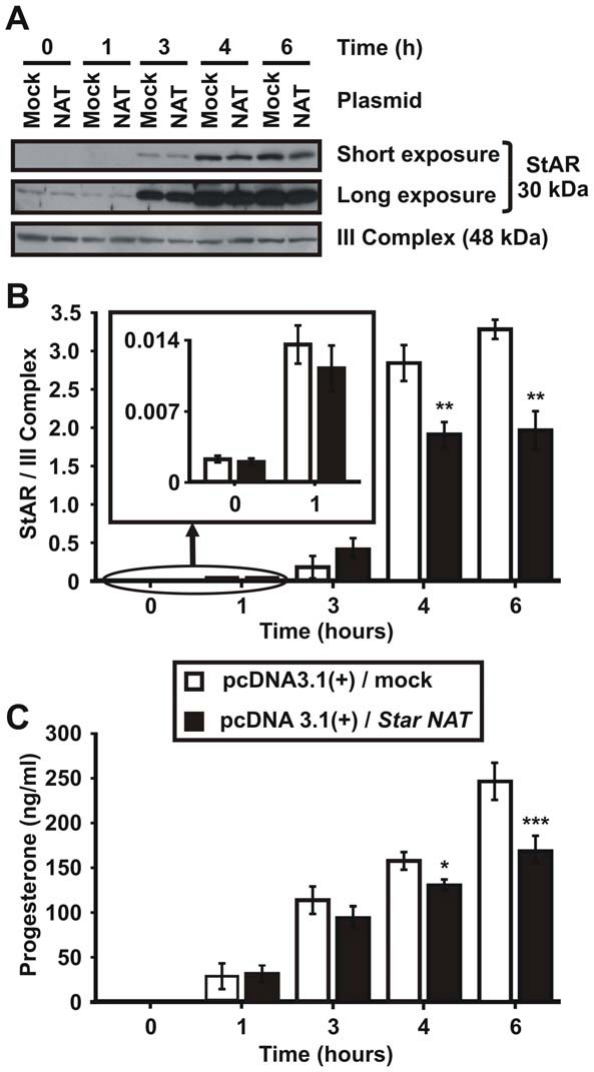
Effect of *Star* NAT overexpression on StAR protein levels and progesterone production. MA-10 cells were transiently transfected with a pcDNA3.1(+) vector expressing *Star* NAT (NAT) or an empty vector (mock) as a control. At 24 h post-transfection, the cells were stimulated with 8Br-cAMP for the indicated times. Mitochondria were isolated from transfected cells and western blotting was performed. **A**. An image of a representative western blot is shown. Membranes were sequentially blotted with anti-StAR and anti-OxPhos complex III core 2 subunit (III Complex) antibodies. Two exposure times are shown for StAR protein expression. **B**. For each band, the OD of the expression level of StAR protein was quantified and normalized to the corresponding III Complex protein. The relative levels of StAR protein are shown. Data are presented as an average ± SD of three independent experiments. *Insert*. Scale amplification for the 0- and 1-h time points. ** *P<*0.01 *vs.* mock-transfected cells stimulated during the corresponding time. **C**. Progesterone concentration in the culture medium was determined by RIA. Results are presented as an average ± SD of four independent experiments. * *P*<0.05 and *** *P*<0.001 *vs.* mock-transfected cells stimulated during the corresponding time.

StAR protein expression was assessed by western blot analysis in mitochondria from transfected cells. *Star* NAT overexpression caused a significant decrease in StAR protein after 4 to 6 h of stimulation (Fig. 9A and B). This result was supported by a concurrent reduction in progesterone production, as determined by radioimmunoanalysis of the cell culture medium (Fig. 9C). Compared to mock-transfected cells, progesterone levels in *Star* NAT-transfected cells decreased 10 to 15% following incubation with 8Br-cAMP for 3 to 4 h. A 30% decrease was observed after 6 h of cAMP stimulation. Altogether these results indicate that, after 4 h of 8Br-cAMP stimulation, *Star* NAT elicits a differential effect on the three *Star* sense transcripts, which ultimately results in reduced StAR protein levels and steroid production. From these results, we conclude that *Star* NAT hormone-regulated expression may be involved in modulating StAR protein expression, a complex process in which post-transcriptional regulation of mRNAs is required.

## Discussion

NATs have been implicated in numerous mechanisms that affect, directly or indirectly, virtually all levels of transcriptional control of eukaryotic gene expression [38], [44].

Here we have identified a new endogenous antisense transcript that we named *Star* NAT because it complements the *Star* mRNA sequence, thus playing a role in the regulation and function of StAR. We have demonstrated its expression in MA-10 Leydig cells and steroidogenic murine tissues. Furthermore, we have established that hCG increases *Star* NAT expression via cAMP. This study constitutes one of a few experimentally proven examples of hormonal regulation of antisense transcripts.

*Star* NAT is 3146-bp long and has full sequence complementarity to the spliced StAR sense 3.5-kb transcript. Similar overlapping and length characteristics have been described for other antisense transcripts [49]. Based on a 200-nucleotide cut-off according to RNA purification protocols, antisense transcripts are classified as short RNAs and long non-protein-coding RNAs [48]. The latter are often several hundred (to thousands) of nucleotides in length and display strict homology to their corresponding sense sequence [38], [50]. While NATs may contain potential ORFs, most are non-coding [35]. We found that *Star* NAT does not translate into any known protein, suggesting that this RNA is most likely a non-protein-coding RNA (ncRNA) [48].

*Star* NAT is polyadenylated since it was efficiently amplified from poly(A)^+^, but not poly(A)^−^, RNA fractions. Moreover, its 3′-end was amplified in a 3′ RACE experiment using an adapter primer which initiates the first strand synthesis at the poly(A) tail of mRNA. Sequencing confirmed the presence of a poly(A) tail in *Star* NAT full sequence. Although many of the NATs found in mice represent atypical transcripts, they tend to be localized in the nucleus and non-polyadenylated [51]. Some NATs are mRNA-like since they possess poly(A) tails and are expressed in the cytoplasm [49], [50] where they may potentially interact with overlapping sense RNAs. It has been suggested that the poly(A) tail is localized to the 3′-end of antisense transcripts, similar to sense transcripts [52]. The presence of a poly(A) tail would confer increased stability to the molecule and thus indicates its localization to the cytoplasm [53].

Three different mechanisms have been proposed to describe the origin of antisense transcripts: 1) antisense synthesis could occur either by transcription of the opposite strand of the corresponding gene (cis-NATs) [54]; 2) transcription of a pseudogene (trans-NATs) [55]; or 3) transcription of the sense mRNA by an RNA-dependent RNA polymerase in the cytoplasm [52], [56]. The mechanism by which *Star* NAT is generated is an interesting question. For instance, this NAT could be produced by transcription from the opposite strand of the *Star* gene. This is consistent with the complementarity of *Star* NAT last 75 bp to *Star* genomic sequence. However, the remaining sequence of *Star* NAT is the complement of the spliced *Star* mRNA. The use of atypical donor and acceptor sites for splicing of the antisense would be required to obtain an exact match between sense and antisense transcripts. Such sites have already been reported and bidirectional transcription has also been proposed [52], [57]. In this case, the precise removal of six introns, as well as a promoter region that regulates *Star* antisense transcription, would be required. However, the current evidence does not support the existence of an origin derived from bidirectional transcription at the gene locus, as has been observed for other NATs [38], [58], [59].

Transcription of the *Star* NAT from a putative spliced pseudogene sequence can be excluded because antisense RNA arising from a pseudogene should exhibit several mutations and therefore display partial complementarity with the sense mRNA [55]. We found only a few point mutations at different positions within the sequence of different clones, which we attributed to classical PCR artifacts produced by the non-proofreading DNA polymerase. Screening the mouse genome database also showed no evidence of a *Star* pseudogene.

Nevertheless, *Star* NAT could be produced by transcription of processed *Star* mRNA in the cytoplasm [60], [61]. This possibility is consistent with the precise and full complementarity of these sense/antisense RNAs. Therefore, an enzyme homologous to RNA-dependent RNA polymerase must be identified [62]. However, direct evidence for such an enzyme in mammalian cells is still lacking [63].

The function of most NATs remains undetermined. Several NATs may constitute transcriptional noise; however, there are clear indications that some have a gene regulatory impact [38], [64]. Furthermore, investigation of the entire antisense transcriptome has identified common structural characteristics of some NATs and point towards conserved themes in gene regulation by antisense transcripts [39], [42], [51].

Expression of NATs in specific tissues provides clues about their physiological role [64]. Analysis of the tissue distribution of *Star* NAT revealed that it is expressed in steroidogenic tissues such as testis, adrenal gland, ovary, and brain, suggesting a role in regulating *Star* sense RNA expression. Comparative expression analysis [65] has demonstrated that, as in our case, NATs tend to be correlated with the expression of the corresponding sense transcript. However, these studies did not specify whether sense and antisense transcripts are expressed within the same cell [66], [67], and frequently this linked expression is not found [68]. Moreover, a hormone-dependent increase in *Star* antisense RNA expression was evidenced by RT-PCR and RPA. Our data show that *Star* NAT expression reached a maximum level at 2 to 3 h after stimulation and indicate that this effect was mediated by cAMP. Our results are among the few recently described examples of effective regulation of NAT expression by hormones [69]-[72]. Sense-antisense *Star* RNA co-expression in MA-10 cells was demonstrated directly by RPA, suggesting that these transcripts could be coordinately regulated. Mammalian RNAs that form sense-antisense pairs have been reported to exhibit reciprocal expression patterns [73]. However, other studies have concluded that NATs display a tendency to be positively correlated with the expression of their sense counterparts [35], [74].

There are several mechanisms by which NATs can influence the expression of their complementary transcripts. These mechanisms have been categorized into four main groups: those related to transcription, RNA–DNA interactions, RNA–RNA interactions in the nucleus, and RNA–RNA interactions in the cytoplasm [34]. Given the results presented here, *Star* NAT could form RNA duplexes with its sense counterpart, which occurs frequently when transcripts are long and completely overlapping [40], [75]. However, this duplex formation has only been detected in a few cases [49], [55], [76] since such hybridization is a complex, transitory, and fragile. Since *Star* NAT appears to be polyadenylated, formation of a cytoplasmic RNA duplex could be postulated as part of its mechanism in regulating StAR expression. Cytoplasmic sense–antisense duplex formation can alter sense mRNA stability and/or translation efficiency, mask protein-binding sites, or generate endogenous small interfering RNA [34].

*Star* is expressed in steroidogenic cells as 3.5-, 2.8-, and 1.6-kb transcripts that differ only in their 3′-UTR, which is derived from alternative polyadenylation sites in exon 7 through the 3′-UTR [20]. In the mouse MA-10 testis and Y-1 adrenal lines, 8Br-cAMP stimulates the *Star* 3.5-kb mRNA preferentially. This level of selectivity has also been observed with adrenocorticotropic hormone stimulation in primary bovine adrenocortical cells. In MA-10 cells, expression of the 3.5-kb mRNA peaks at 3 h but then declines rapidly, whereas the 1.6-kb mRNA is maintained at a steady-state level after 6 h of stimulation [24], [29], [77]. The 3.5-kb *Star* mRNA is intrinsically much less stable when expressed from vectors but is similarly translated [19]. This differential timing in expression of the 3.5- and 1.6-kb *Star* transcripts is most likely due to differences in post-transcriptional regulation. The time course of *Star* NAT expression after hormone stimulation nearly paralleled that of the *Star* 3.5-kb sense transcript. After achieving peak levels 2 to 3 h after stimulation, *Star* antisense RNA levels declined. In addition, we did not observe any changes in the expression of longer sense transcript levels after *Star* NAT overexpression, suggesting that the antisense transcript may play a role in stabilizing the 3.5-kb *Star* mRNA in the cytoplasm. The overlapping region may affect mRNA stability by altering its secondary or tertiary structure or by interacting with mRNA decay regulatory proteins, such as AURE-binding proteins. Interestingly, the 3.5-kb *Star* mRNA possesses an AURE within the extended 3′-UTR. The zinc finger protein ZFP36L1/TIS11b binds to UAUUUAUU repeats in the extended 3′-UTR, enhancing *Star* mRNA turnover. Surprisingly, TIS11b expression is induced concurrently with *Star* 3.5-kb mRNA. This co-regulation, despite an adverse affect on mRNA stability, may be explained by the presence of *Star* NAT [29]. *Star* antisense RNA could mask the AURE motifs required for TIS11b-mediated destabilization. Indeed, the antisense transcript overlaps the region that contains the first two UAUUUAUU repeats shown to be necessary for enhanced turnover. Therefore, cAMP could increase *Star* 3.5-kb mRNA stability by concomitantly increasing *Star* NAT expression, thus allowing for rapid StAR protein synthesis and cholesterol transport. Later, *Star* antisense transcript levels could decline, enabling destabilizing factors (e.g., AURE-dependent or -independent) to promote mRNA decay. An increase in mRNA stability provides a means to quickly respond to stimulation by cytokines, growth factors, and protooncogenes [78], [79].

Alternatively, longer 3′-UTRs frequently possess target sites for miRNAs, which mediate mRNA degradation [31]. Many NATs may have the ability to mask miRNA-binding sites following cytosolic RNA duplex formation [34]. Although the impact of miRNAs on *Star* expression has yet to be examined, one prospective miRNA (mmu-miR-706) site has already been identified within the 3′-UTR of rodent *Star* mRNA [19], [32]. The *Star* NAT sequence also overlaps this region, implying that a miRNA-related mechanism may be involved.

Overexpression of *Star* NAT resulted in an increase in 2.8- and 1.6-kb *Star* mRNAs with a concomitant decrease in StAR protein. Although much of the mechanism remains to be elucidated, we can postulate that it does not involve an AURE-dependent increase in stability because *Star* 2.8- and 1.6-kb transcripts lack this sequence within their 3′-UTR. Since low levels of StAR protein were observed despite high levels of the mid-and short-length mRNAs, these transcripts may not be actively translated, but instead could be playing a role in regulating expression of the 3.5-kb mRNA.

Differences in how *Star* NAT and sense transcripts interact may be due to variations in their secondary and tertiary structures, as well as on the complexity of forming RNA duplexes [80]. The exact mechanisms underlying the possible functional role of *Star* NAT in the cAMP signal transduction pathway remain to be investigated. Nevertheless, our data indicate that regulation of StAR protein levels may depend on the presence of a natural antisense transcript. Hormonal regulation of this transcript and its role in balancing the expression of a crucial protein involved in cholesterol transport emphasize the importance of these results. Moreover, the involvement of NATs provides an additional mechanism for rapid hormonal regulation of steroidogenesis.

Regulation of StAR expression is a complex process involving transcriptional and post-transcriptional mechanisms. Our work establishes the concept that StAR protein levels are modulated by a fine balance between sense and antisense transcripts that enables a quick response to hormonal stimulation. Participation of the *Star* NAT in this process may explain the preferred formation of a longer *Star* transcript, which facilitates the rapid synthesis of StAR protein and cholesterol transport after hormone stimulation. In conclusion, the present results demonstrate, for the first time, that antisense transcription adds another level of control to gene expression in steroid synthesis regulation.

## Materials and Methods

### Ethics statement

All animal experiments were performed according to the guidelines recommended by the National Institute of Health and protocols approved by the Institutional Ethical Committee from the School of Medicine, University of Buenos Aires (approval ID 1072/05 and 1347/06 CD, School of Medicine).

### Computational analysis

The murine *Star* gene (Gene ID: 20845) was aligned with a mouse EST database using BLAST [81]. The results were visualized using the UCSC Genome Browser [45]. Homologous sequences were compared against the murine cDNA derived from the longest *Star* mRNA (NM_011485) and analyzed using the Unigene database (NCBI), Blast2sequence (NCBI), and the Vector NTITM Suite 8 (InforMax, Inc.) software. The latter program was also used to identify open reading frames (ORFs) and the identity of all sequenced fragments. A potential promoter region for *Star* NAT transcription was evaluated using PROSCAN version 1.7 (bimas.dcrt.nih.gov/molbio/proscan).

### Materials

8Br-cAMP, yeast tRNA, and ribonuclease A (RNase A) were purchased from Sigma-Aldrich (St. Louis, MO). Waymouth MB752/1 cell culture media, Opti-MEM and LipofectamineTM 2000 were obtained from Gibco-Life Technologies Inc. (Carlsbad, CA). M-MLV reverse transcriptase (RT), T7 RNA polymerase, GoTaq® DNA polymerase, pGEM®-T Easy vector, RNAsin® inhibitor, RNase-free DNase RQ1, and other molecular biology reagents were purchased from Promega (Madison, WI). RNase-free Deoxyribonuclease I (DNase I) Amplification Grade, the pcDNA3.1(+) vector, and oligonucleotides were obtained from Invitrogen (Carlsbad, CA). Proteinase K was purchased from Roche Diagnostics (Buenos Aires, Argentina). All other reagents were of the highest grade available.

### Mammalian cell culture and animals

The MA-10 cell line is a clonal strain of mouse Leydig tumor cells that produces progesterone rather than testosterone as the main steroid. MA-10 cells were generously provided by Mario Ascoli from the University of Iowa, College of Medicine (Iowa City, IA) and were handled as described previously [82]. The growth medium consisted of Waymouth MB752/1 containing 1.1 g/l NaHCO_3_, 20 mM HEPES, 50 g/ml gentamicin, and 15% heat-inactivated horse serum. Flasks and multiwell plates were maintained at 36°C in a humidified atmosphere containing 5% CO_2_.

Human chorionic gonadotropin (purified hCG, batch CR-125 of biological potency 11900 IU/mg; gift from NIDDK, NIH) was used to treat the MA-10 cells (20 ng/ml) for the times indicated. 8Br-cAMP (Sigma-Aldrich, St. Louis, MO) a permeable analog of cAMP, was used to treat the MA-10 cells (1 mM) for the times indicated.

According to approved protocols of animal care and use, tissues were obtained from 60-day-old male and female Balb/c mice (purchased from the School of Pharmacy and Biochemistry, University of Buenos Aires, Buenos Aires, Argentina).

### Total and poly(A)^+^ RNA isolations

Total RNA from MA-10 cells and Balb/c tissues was isolated with Tri-Reagent (Molecular Research Center, Inc. Cincinnati, OH) according to the manufacturer's instructions. Poly(A)^+^ and poly(A)^−^ RNA from MA-10 cells were fractionated by oligo(dT) cellulose chromatography as published [83]. RNA concentration was quantitated in triplicate. Any residual genomic DNA was removed by treating RNA with DNase I (15 min at room temperature), which was subsequently inactivated by incubation with 2.5 mM EDTA for 10 min at 65°C.

### 5′ RACE

5′ RACE was performed using the SMARTTM RACE cDNA Amplification kit (Clontech Laboratories, Inc., Mountain View, CA) according to the manufacturer's instructions. Three different groups of three sequence-specific primers were used for the RT, PCR, and nested PCR analysis. A list of the oligonucleotides used is shown in Table 1. Total DNase I-treated RNA was reverse-transcribed using the first sequence-specific primer of each group. PCR and nested PCR amplifications were conducted with the two remaining oligonucleotides of each group. PCR conditions were: one cycle at 94°C for 5 min; 5 cycles of 94°C for 30 s, 70°C for 30 s, 72°C for 3 min; 5 cycles of 94°C for 30 s, 68°C for 30 s, 72°C for 3 min; 25 cycles of 94°C for 30 s, 66°C for 30 s, 72°C for 3 min; and one cycle at 72°C for 10 min. PCR products were resolved on 1.5% (wt/vol) agarose gels containing 0.5 µg/ml of ethidium bromide to determine the molecular size of the amplicons. Bands were visualized by UV transillumination and images digitally recorded. Bands were excised, eluted, and ligated into the pGEM®-T Easy vector, and then sequenced using T7/SP6 primers and the ABI 3700 sequencer (Applied Biosystems-Life Technologies, Carlsbad, CA). Results were analyzed using BLAST and Vector NTITM Suite 8 software.

### 3′ RACE

3′ RACE was performed using the Invitrogen kit (Carlsbad, CA) according to the manufacturer's instructions. Total DNase I-treated RNA was reverse-transcribed at 50°C using the adapter primer (AP) which initiates the first strand synthesis at the poly(A) tail of mRNA. PCR and nested PCR amplifications were conducted with two sequence-specific primers and the two universal amplification primers provided with the kit. A list of the oligonucleotides used is shown in Table 1. PCR conditions were: 1 cycle at 94°C for 5 min; 5 cycles at 94°C for 30 s, 64°C for 30 s, 72°C for 3 min; 5 cycles at 94°C for 30 s, 62°C for 30 s, 72°C for 3 min; 30 cycles at 94°C for 30 s, 60°C for 30 s, 72°C for 3 min; and 1 cycle at 72°C for 10 min. PCR products were resolved on 1.5% (wt/vol) ethidium bromide-stained agarose gels to determine the molecular size of the amplicons. Bands were excised, eluted, and sequenced. Results were analyzed using BLAST and Vector NTITM Suite 8 software.

### RT-PCR and nested PCR amplifications

RNA was reverse-transcribed with M-MLV reverse transcriptase and G3.1 or Rv.RT *Star* NAT-specific primers. RT reactions were carried out at high temperature (50°C) to avoid primer-independent cDNA synthesis [47] and to improve the strand specificity of RT-PCR detection.

PCR amplification was performed using GoTaq® DNA polymerase with the pair of primers listed in the corresponding section of Table 1. Amplification conditions were the same as those employed for 5′ or 3′ RACE assays. PCR products were resolved on 1.0% (wt/vol) ethidium bromide-stained agarose gels, excised, eluted, and finally cloned into a pGEM®-T Easy vector for sequencing. As mentioned previously, RNA was treated with RNase-free DNase I to eliminate genomic DNA contamination. In addition, several negative controls were added. First, an RNase-treated RNA control was included to determine whether any nucleic acid contamination was present in the reaction. Second, DNase-treated RNA was subjected to RT-PCR without reverse transcriptase to ensure the lack of contaminants and effectiveness of DNase I treatment. Finally, two controls were conducted in order to asses the strand specificity of reverse transcription: RT reactions were carried out in either the absence of primers, or in the presence of a non-specific primer that is not related to the target sequence. The latter targets the murine *acyl-CoA synthetase long-chain family member 4* mRNA. As expected, no PCR product was detected from these negative control reactions.

### Semi-quantitative RT-PCR

For semi-quantitative RT-PCR of *Star* NAT (amplicon size 460 bp), sequence-specific RT was performed using the G3.1 primer, followed by PCR amplification using the primers in Table 1. L19 ribosomal protein mRNA (amplicon size 405 bp) was used as internal standard to compare amplified amounts of *Star* NAT from different RNA samples. In this case, the RT reaction was conducted using random primers. PCR conditions were one cycle at 94°C for 5 min, followed by 27 (for *Star* NAT) or 23 cycles (for *L19*) of 94°C for 30 sec, 50°C for 30 sec, 72°C for 1 min, and one cycle at 72°C for 10 min. The number of cycles used was optimized for each transcript to fall within the linear range of PCR amplification.

For semi-quantitative RT-PCR of *Star* sense transcripts, sequence-specific reactions were also performed. Since the three StAR mRNAs differ only in their 3′-UTR, it was difficult to discriminate between them. Thus, three primer pairs targeting different regions within mouse *Star* mRNA were used (Table 1). One pair (Sense 1, amplicon size 435 bp, 22 cycles) was designed to target only the extended 3′-UTR of the longest 3.5-kb mRNA. A second pair (Sense 2, amplicon size 719 bp, 23 cycles) targeted a region that is shared by both 2.8- and 3.5-kb *Star* mRNAs. A third pair (Sense 3, amplicon size 566 bp, 21 cycles) was specific to the coding region and was thus common to all three *Star* sense transcripts (1.6-, 2.8- and 3.5-kb). PCR conditions were the same as described above. PCR products were resolved on 1.5% (wt/vol) ethidium bromide-stained agarose gels to determine the molecular size of the amplicons. Bands were visualized and transcript levels quantitated using the computer-assisted image analyzer Gel-Pro (IPS, North Reading, MA). Bands were excised, eluted, and cloned into the pGEM®-T Easy vector for sequencing.

### *In vitro* transcription of RNA probes

Single-chain *Star* sense and *Star* NAT [^32^P]-labeled riboprobes were synthesized by *in vitro* transcription. The *Star* NAT RNA probe (NAT probe 1, 779 bp) was complementary to 719 bp of *Star* NAT sequence. The template for its synthesis was a pGEM®-T Easy vector containing a *Star* sense fragment amplified by semi-quantitative RT-PCR using the Sense 2 primer pair and cloned in the sense position relative to the *T7 RNA polymerase* promoter. The *Star* sense RNA probe (sense probe, 626 bp) was complementary to 566 bp of the coding region of sense transcripts. The template for its synthesis was a pGEM®-T Easy vector containing a *Star* sense fragment amplified by semi-quantitative RT-PCR using the Sense 3 primer pair and cloned in the antisense position relative to the *T7 RNA polymerase* promoter. Both plasmids were linearized with *Sal* I and gel-purified. Another *Star* NAT probe (NAT probe 2, 680 bp) was synthesized to be complementary to 590 bp of the *Star* NAT sequence in a region different from the previous NAT probe used. The template for its synthesis was the *Star* cDNA sequence cloned into a pGEM®-T Easy vector in the sense position relative to the *T7 RNA polymerase* promoter as previously generated in our laboratory [84]. This plasmid was linearized with *Xho* I and gel-purified. The choice of probes size (779-, 626- and 680-bp) was conducted according to the recommendations for RPA methodology [85]. Riboprobe synthesis reactions contained 0.5 -1 µg of linearized DNA plasmid; ATP, GTP, CTP (0.5 mM each); 12 µM UTP; 100 µCi [α^32^P] UTP (3000 Ci/mmol specific activity from New England Nuclear, Boston, MA); 5 mM dithiothreitol; 20 U RNAsin® inhibitor; 20 U T7 RNA polymerase; and 1X transcription buffer. After 2 h at 37°C, DNA templates were digested with RNase-free DNase RQ1. Transcription products were resolved on 5% denaturing polyacrylamide gels (8 M urea). Gel slices containing full-length transcripts were excised and incubated overnight at 37°C in gel elution buffer (0.5 M ammonium acetate, 1 mM EDTA, 0.1% SDS). The concentration of RNA probes was 3–6×10^4^ cpm/µl, with a theoretical specific activity of 3×10^8^ cpm/µg.

### RNase Protection Assay (RPA)

Probes (sense and NAT 1 probes or NAT 2 probe alone, 72×10^4^ cpm each) and DNase I-treated total MA-10 cells RNA (80 µg) were co-precipitated and hybridized overnight at 42°C in hybridization buffer (40 mM PIPES pH 6.4; 0.5 M NaCl, 1 mM EDTA, 75% formamide). Then, RNase A (40 µg/ml) digestion was performed for 1 h at 32°C. The reaction was stopped by adding 0.5% SDS, followed by digestion with 12.5 mg/ml proteinase K at 37°C for 15 min. Next, a phenol/chloroform extraction was performed by adding 10 µg yeast tRNA to each sample as carrier. After ethanol precipitation, protected double-chain RNA fragments were subjected to a 5% denaturing polyacrylamide gel (8 M urea) chromatography and then visualized by autoradiography. The negative control (tRNA(+)RNase) consisted of yeast tRNA instead of MA-10 RNA. No bands are expected in this sample following digestion. The tRNA(−)RNase control consisted of yeast tRNA without ribonuclease treatment and was used to visualize the full-length probes.

### Plasmid preparation and *Star* NAT overexpression by transient transfection

*Star* NAT sequence (2757-bp length, obtained from RT-PCR amplification based on 5′RACE data), was subcloned from a pGEM®-T Easy vector into the eukaryotic expression vector pcDNA3.1(+) using *Not* I restriction sites.

For transient transfection, MA-10 cells were seeded the day before and grown up to 80% confluence. Transfection was performed with a pcDNA3.1(+) plasmid expressing *Star* NAT or an empty vector in Opti-MEM medium and LipofectamineTM 2000 reagent according to the manufacturer's instructions. Transfection efficiency was estimated at approximately 30% by counting fluorescent cells transfected with the pRc/CMVi plasmid expressing enhanced green fluorescent protein (EGFP) [84].

### Northern blotting

Samples of total RNA (24 µg) were resolved on 1.2% agarose/2.2 M formaldehyde gels and transferred onto Hybond-N+ nylon membranes (Amersham Biosciences, Stockholm, Sweden). A single-strand *Star* sense RNA probe was synthesized by in vitro transcription as we detailed previously here. After prehybridization for 6 h at 56°C, blots were hybridized at 56°C overnight with [^32^P]-labeled riboprobe. The hybridization solution contained 6X SSPE, 5X Denhardt's solution, 0.5% formamide, 10 ng/ml yeast tRNA, and 100 µg/ml denatured salmon sperm DNA. Blots were subsequently washed twice with 2X SSPE (150 mM NaCl, 10 mM NaH_2_PO_4_, 1 mM EDTA)/0.5% SDS at room temperature; twice with 1X SSPE/0.1% SDS at 65°C; and twice with 0.1X SSPE/0.1% SDS at 65°C. StAR hybridization signals were detected using a Storm PhosphorImager (Amersham Biosciences, Stockholm, Sweden) and normalized to 18S RNA levels.

### Protein quantification and western blotting

Mitochondria were isolated as described previously [86]. Mitochondrial proteins were determined by the Bradford assay [87] using BSA as a standard. Proteins were separated by SDS-PAGE (12% acrylamide gels) and transferred to PVDF membranes (Bio-Rad Laboratories Inc., Hercules, CA) as described previously [84]. Anti-StAR antibody was generously provided by Dr. Douglas Stocco (Texas Tech University Health Sciences Center, Lubbock, TX). Membranes were sequentially blotted with anti-StAR and anti-OxPhos complex III core 2 subunit (Invitrogen, Carlsbad, CA) antibody, and immunoreactive bands were detected using enhanced chemiluminescence (GE Healthcare, Buckinghamshire, UK).

### Radioimmunoanalysis (RIA) and statistics

Progesterone production in cell culture media was measured by RIA as described previously [88]. Data are represented in ng/ml. Statistical significance was determined using the Student's t test or analysis of variance (ANOVA) followed by the Student-Newman-Kuels test.

## Supporting Information

Figure S1 **Genomic view of** ***Star*** **gene related RNA and EST sequences.** The potential antisense transcripts (NATs predicted *in silico*) are highlighted with a pink circle in front of the sequence name.
(PPT)
