# Supplementary material for: Hormone-Dependent Expression of a Steroidogenic Acute Regulatory Protein Natural Antisense Transcript in MA-10 Mouse Tumor Leydig Cells
Source: PLoS One. 2011 Aug 1;6(8):e22822. doi: 10.1371/journal.pone.0022822 (PMC3148237; doi:10.1371/journal.pone.0022822)
Supplement: Figure S1 — Genomic view of Star gene related RNA and EST sequences. The potential antisense transcripts (NATs predicted in silico) are highlighted with a pink circle in front of the sequence name. (PPT) [file pone.0022822.s001.ppt]

## Slide 1
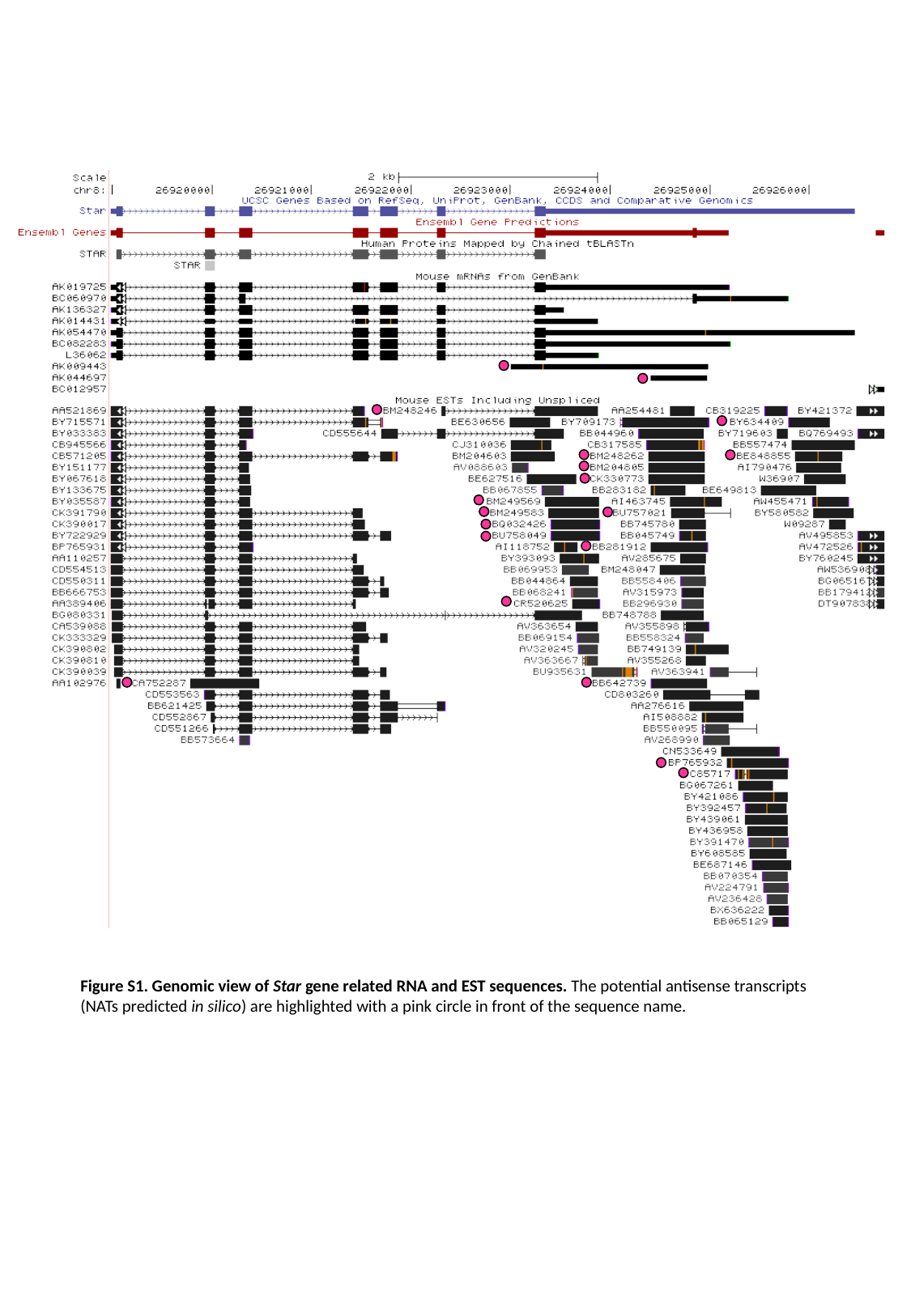

Figure S1. Genomic view of Star gene related RNA and EST sequences. The potential antisense transcripts (NATs predicted in silico) are highlighted with a pink circle in front of the sequence name.
